# AOD: the antioxidant protein database

**DOI:** 10.1038/s41598-017-08115-6

**Published:** 2017-08-07

**Authors:** Pengmian Feng, Hui Ding, Hao Lin, Wei Chen

**Affiliations:** 1 Hebei Province Key Laboratory of Occupational Health and Safety for Coal Industry, School of Public Health, North China University of Science and Technology, Tangshan, 063000 China; 2 Key Laboratory for Neuro-Information of Ministry of Education, School of Life Science and Technology, Center for Informational Biology, University of Electronic Science and Technology of China, Chengdu, 610054 China; 3 Department of Physics, School of Sciences, and Center for Genomics and Computational Biology, North China University of Science and Technology, Tangshan, Tangshan, 063000 China

## Abstract

An antioxidant is a molecule that can prevent free radicals from causing damages in organisms. The increasing studies on antioxidants calls for a specialized database that is not readily available yet. To this end, in the present study, the Antioxidant Database (AOD) was developed to help researchers understand and reveal the biological functions of antioxidant proteins. AOD is freely available at http://lin.uestc.edu.cn/AODdatabase/index.aspx. The current release of AOD consists of 710 antioxidant proteins. Information including taxonomy, source organism, subcellular location, gene ontology, catalytic activity and function of antioxidant proteins are all extracted from UniProtKB/Swiss-Prot and captured in AOD. In addition, two web-based tools for performing sequence similarity search and computationally identification of antioxidants were also integrated in AOD. We believe that AOD will greatly facilitate the researches on antioxidants.

## Introduction

An antioxidant, also known as the free radical scavenger, is a molecule that can neutralize free radicals and thus preventing them from causing damages^1^. Recent studies have demonstrated that antioxidants play important roles in the management or prevention of cancers^2^, coronary heart disease^3^, macular degeneration^4^, Alzheimer’s disease^5^, arthritis-related conditions^6^, and longevity^7^. Therefore, antioxidants have attracted considerable attentions of scientists who focus on cancer prophylaxis and therapy and human health.

Our human body naturally produces antioxidants to counteract the damaging effects of free radicals^8^. Endogenous antioxidants are enzymes, such as superoxide dismutase^9^, catalase^10^, glutathione peroxidase^11^ or nonenzymatic compounds including uric acid^12^, bilirubin^13^, metallothioneins^14^. However, the amount of free radicals is often greater than that of the naturally occurring antioxidants in organisms. In order to balance the disequilibrium, it’s necessary to obtain antioxidants from external sources.

In the past several decades, multiple types of exogenous antioxidant proteins have been detected, such as vitamin A, vitamin C, and vitamin E^15, 16^, and a huge amount of studies about antioxidants have also been reported. However, there isn’t a database systematically collecting and compiling the information of antioxidants at present. Therefore, it is urgent need to develop a database, where the researchers could obtain the precise information of known antioxidant proteins.

The enormous amount of data on antioxidants had motivated us to develop a general database. Hence, in the present study, we established the antioxidant database (AOD) with an objective to provide useful insights to the study of antioxidants.

## Results and Discussions

### Data statistics

**Figure 1:**
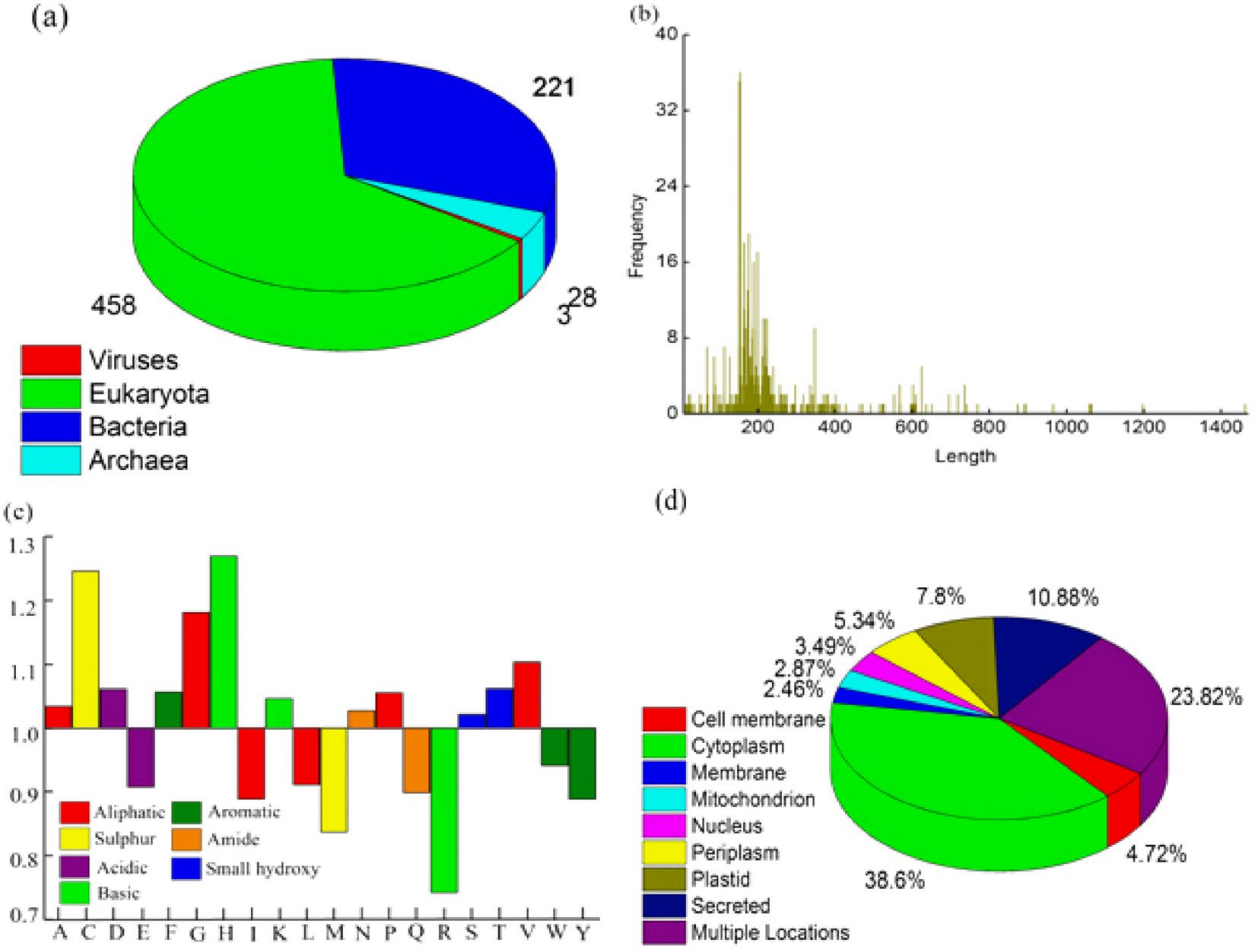
The statistical analysis of AOD. (**a**) Organism distribution of antioxidant proteins in AOD; (**b**) Length distribution of antioxidant proteins in AOD; (**c**) Relative amino acid composition of antioxidant proteins in AOD as compared with Swiss-Prot composition; (**d**) Subcellular location distribution of antioxidant proteins in AOD.

Among the 710 antioxidant proteins of AOD, 458 are from eukaryota, 221 from bacteria, 28 from archaea and 3 from virus (Fig. 1a). In AOD, 94% of the antioxidant proteins contain more than 100 amino acids with an average length of 227. The longest antioxidant protein (A1IGV8) has 1,463 amino acids and the shortest one (P83233) merely 8 amino acids. The length distribution of antioxidant proteins in AOD is shown in Fig. 1b.

The ratio of the frequency of amino acids in AOD to the frequency of amino acids in UniProtKB/Swiss-Prot protein knowledgebase is shown in Fig. 1c. We found that antioxidant proteins are enriched in residues Cys, Gly, His and Val.

The subcellular location of antioxidant proteins is closely correlated with its biological activities^17^. Therefore, the information about subcellular location of antioxidant proteins is also provided in AOD. Four hundred and eighty seven antioxidant proteins in AOD have annotated subcellular location information, and 76% of them only reside in a single subcellular localization and the other 24% in multiple subcellular locations (Fig. 1d).

### Quality assessment

In order to evaluate the quality of AOD, the 710 antioxidant proteins in the database were manually checked. It was found that there are 609 antioxidant proteins that have been reported in previous studies as indicated in the UniProtKB/Swiss-Prot. The Uniprot IDs for these 609 proteins were listed in Supplementary Table S1. As indicated in UniProtKB/Swiss-Prot, the remaining 101 antioxidant proteins were evaluated by experimental methods at transcript level, which supports the existence of the protein.

### Browse

By clicking the ‘Browse’ button, the antioxidant proteins in AOD database will be shown on the computer screen and can be displayed page by page. The antioxidant proteins could be selected by clicking the buttons on their left. The information of protein name, taxonomy, organism, subcellular location, gene ontology, catalytic activity, function, amino acid sequence and links to external database such as Swiss-Model Repository, Uniprot, Gene Ontology and NCBI for each entry can be viewed by clicking on the sequence ID or by clicking the ‘Show’ button on the top right corner. The selected antioxidant proteins could also be downloaded to local machine by clicking the ‘Download’ button and saved in fasta format.

### Search

The AOD can be searched in multiple ways. Users can basically search antioxidant proteins by limiting the searching filed to UniProt ID, Taxonomy, Organism, Subcellular locations or Protein names and entering the related query keyword. To perform conditional search, user can add (or remove) the searching filed using the Add (or Remove) button and then join the multiple query keywords by logical operators like AND/OR.

### Tools

AOD integrates two web-based tools for performing further analyses, i.e. sequence similarity search and identification of antioxidants. We have integrated BLAST^18^ in AOD that allows users to perform the BLAST search against sequences deposited in AOD, which will facilitate finding sequences in the database that have high sequence similarity with the query sequence.

To the best of our knowledge, **AodPred** ^19^ is the smartest computational tool for identifying antioxidants at present. For the convenience of experimental scientists, the **AodPred** predictor was also integrated in AOD. Users can employ it to predict whether a query protein sequence is antioxidant or not. After inputting the query sequences in fasta format and clicking the ‘Submit’ button, the probability of the predictions being antioxidant or non-antioxidant will be shown in a new page.

## Conclusions

In the present study, a comprehensive database called AOD is built with the aim to provide useful insights to the study of antioxidant proteins and to help researchers understand the role of different properties of antioxidant in their antioxidative activities. AOD is the first database providing information on antioxidants from multiple perspectives. We hope the AOD will better serve the research on antioxidant proteins. In order to make an encyclopedia-like database for antioxidants, we will continue to accumulate the information of new antioxidant proteins and add them into AOD.

## Methods and Materials

### Data collection

Sequences of antioxidant proteins together with their information including taxonomy, source organism, subcellular location, gene ontology, catalytic activity and function were obtained from the UniProtKB/Swiss-Prot database (release 2016_11)^20, 21^ by searching the keyword “antioxidant”. In order to obtain the quality data, the following steps were performed: (i) only proteins with the experimentally confirmed antioxidative activities were included; (ii) proteins including illegal letters, i.e., “B”, “X” or“Z”, were excluded. This finally yields 710 proteins that have experimentally proven or confirmed antioxidative activity to be included into AOD.

### Database structure and interface

As a user-friendly database, AOD is freely available at http://lin.uestc.edu.cn/AODdatabase/index.aspx. The database main page contains the following interfaces: Home, Browse, Search, Tools, Statistics, Links and Contact. The information related to protein name, taxonomy, origin of organism, subcellular location, sequence length, gene ontology, catalytic activity, protein sequence, function and links to external database such as Swiss-Model Repository, Gene Ontology and NCBI are all provided in AOD.

## Electronic supplementary material

Supplementary Table S1
