# Supplementary material for: AOD: the antioxidant protein database
Source: Sci Rep. 2017 Aug 7;7:7449. doi: 10.1038/s41598-017-08115-6 (PMC5547145; doi:10.1038/s41598-017-08115-6)
Supplement: Supplementary file 1 — Supplementary Table S1 [file 41598_2017_8115_MOESM1_ESM.pdf]

Supplementary Materials

AOD: the antioxidant protein database

Pengmian Feng[1], Hui Ding[2], Hao Lin[2]*, Wei Chen[3]*

[1] Hebei Province Key Laboratory of Occupational Health and Safety for Coal Industry, School of Public Health, North China University of Science and Technology, Tangshan 063000, China;
[2] Key Laboratory for Neuro-Information of Ministry of Education, School of Life Science and Technology, Center for Informational Biology, University of Electronic Science and Technology of China, Chengdu 610054, China;
[3] Department of Physics, School of Sciences, and Center for Genomics and Computational Biology, North China University of Science and Technology, Tangshan, Tangshan 063000, China.

***To whom correspondence should be addressed.**

Hao Lin: hlin@uestc.edu.cn
Wei Chen: greatchen@ncst.edu.cn;
Tel: +86-315 3725715; Fax: +86-315 3725715

Supplementary Table S1 The Uniprot ID of the antioxidant proteins in AOD that have been reported in previous studies.

A0PSD4	O04996	O74887	P0A251	P14630	P28757	P49114	P66952
A0QQJ4	O04997	O75444	P0A252	P14830	P28758	P49537	P66953
A0R1V9	O08709	O77834	P0A4M6	P14831	P28782	P49908	P66954
A0RBT0	O08807	O78310	P0A5N4	P15107	P28783	P49924	P69049
A1IGV8	O08997	O82089	P0A5N5	P15453	P29427	P50115	P70195
A1KLC4	O09164	O94561	P0A608	P16026	P29428	P50116	P70274
A2BJD9	O12933	O95445	P0A862	P18122	P29895	P50117	P80174
A2QMY6	O13851	P00441	P0A863	P18846	P30041	P51272	P80239
A2XGP6	O14463	P00442	P0A864	P19006	P30044	P51547	P80566
A3DKL1	O19097	P00443	P0A865	P19007	P30048	P52552	P80732
A4IQF5	O22287	P00445	P0A866	P19440	P31307	P52571	P80734
A4YYT0	O22373	P00446	P0A9Q7	P19476	P31308	P52572	P80735
A5ENR3	O22711	P00450	P0AE08	P19880	P31725	P52574	P80740
A5F3A2	O24364	P00738	P0AE09	P20108	P32119	P53615	P80864
A6X5N3	O25151	P02649	P0AE10	P20379	P33431	P54407	P81036
A8A9P0	O26262	P02650	P0AE11	P20735	P34227	P54783	P81082
A8L1J2	O29969	P02662	P0AE52	P21762	P34461	P54843	P81163
A8XCP3	O31687	P02666	P0AGD1	P22217	P34697	P54844	P81269
A9KBI4	O31699	P02692	P0AGD2	P22233	P34760	P55157	P81361
B0M2T2	O31820	P02768	P0C0L2	P22803	P34936	P56597	P81926
B1W2G7	O34564	P03946	P0C0L3	P23345	P35160	P56876	P82205
B2HD59	O34777	P04178	P0C2J8	P23346	P35700	P57005	P83129
B2SAW2	O35086	P05067	P0C5C8	P23417	P35704	P57279	P83200
B3EWR3	O35244	P06702	P0C5C9	P24669	P35705	P57668	P83233
B6IZ19	O42666	P06727	P0C5D0	P24702	P36077	P57790	P83684
B8GVX4	O42724	P06866	P0C5D1	P24704	P36214	P57880	P84729
C1AQZ5	O43091	P07148	P0C5D4	P24705	P38013	P59760	P85278
C1F4K1	O43283	P07314	P0C5D5	P24706	P38918	P59960	P85978
C3PGV9	O46412	P07505	P0C6P9	P24707	P40553	P60052	P86215
C4LJ26	O54968	P07509	P0CB50	P25236	P40554	P61851	P86241
D0EYG3	O55234	P07632	P0CV91	P25325	P40581	P61852	P86919
D0ZW85	O58966	P08226	P0CW86	P25713	P41962	P61854	P91883
D2HKB0	O59858	P08228	P10791	P25788	P41963	P63300	P91938
E7FB98	O59924	P08294	P10792	P25842	P41973	P63301	P93407
E8XDJ8	O65768	P09212	P11418	P26830	P42365	P63302	P95855
G3ETQ2	O66602	P09670	P11428	P27005	P42366	P63303	P95895
G3ETQ3	O66780	P09678	P11964	P27082	P43787	P63345	P97532
G3F828	O67024	P09936	P12710	P27170	P46309	P65633	P99029
O00244	O67149	P0A0B5	P13635	P28343	P46418	P66004	P99074
O00522	O68901	P0A0B6	P13926	P28492	P48506	P66826	P99146
O04005	O69649	P0A0B7	P14312	P28755	P48822	P66827	P99999

Supplementary Table S1 continued						
Q00637	Q49126	Q5VAN1	Q6URB0	Q8HXQ4	Q96A56	Q9M7T0
Q01137	Q49UT8	Q5YT53	Q71Z84	Q8K9W0	Q96VL0	Q9MB35
Q02610	Q49YE4	Q5ZI34	Q72NR4	Q8KBN8	Q96XS5	Q9N0V4
Q02BZ5	Q4FP21	Q5ZJF4	Q73B22	Q8KED5	Q974S8	Q9NL98
Q03247	Q4L376	Q5ZKS8	Q73RS4	Q8L5E0	Q979N7	Q9PPE0
Q04120	Q4L754	Q5ZT17	Q73ZL4	Q8LBK6	Q97E14	Q9QXE4
Q04JB8	Q500Y9	Q60692	Q74NC6	Q8N573	Q98SV0	Q9R063
Q06830	Q50232	Q60795	Q751L8	Q8NRG3	Q98SV1	Q9R1P4
Q06892	Q50416	Q60928	Q75SY5	Q8NW51	Q99497	Q9RU48
Q07182	Q50441	Q61147	Q7AKI6	Q8PYP6	Q99J99	Q9SDD6
Q07449	Q51853	Q61171	Q7BHK8	Q8R844	Q99LX0	Q9SRZ4
Q07796	Q54G70	Q61646	Q7F8S5	Q8RCY5	Q99MH5	Q9TT99
Q08420	Q54GP3	Q61985	Q7G959	Q8SPS7	Q99MS3	Q9UI32
Q0BUC5	Q54PZ2	Q63716	Q7M1R5	Q8SS85	Q9A268	Q9URV9
Q0BXT2	Q54RN1	Q63DQ8	Q7RTV5	Q8U218	Q9AGW2	Q9USR1
Q0D840	Q54RQ1	Q64405	Q7XPE8	Q8VWJ1	Q9ANL0	Q9UT98
Q0DRV6	Q54TU5	Q65HX8	Q80YE2	Q8WNN6	Q9AU11	Q9UZV4
Q0RH96	Q54TW8	Q684M4	Q81FU5	Q8XVP0	Q9BQE4	Q9V3P0
Q10252	Q55060	Q694A3	Q81SZ9	Q8Y6U8	Q9BRX8	Q9VNT5
Q10801	Q555L5	Q69TY4	Q82IC5	Q8YCF2	Q9BYN0	Q9VX10
Q12548	Q55624	Q6AXX6	Q83BM5	Q8Z7A8	Q9C0N4	Q9WUC4
Q13162	Q55GQ5	Q6B4U9	Q877B5	Q8ZE42	Q9C5R8	Q9WZR4
Q14145	Q56461	Q6C662	Q89AS1	Q8ZP65	Q9CBI6	Q9X5V1
Q15165	Q56YN3	Q6CPE2	Q8AV19	Q8ZPS6	Q9CR58	Q9XEX2
Q16236	Q57109	Q6EEV6	Q8CMQ2	Q90023	Q9CYH2	Q9XT28
Q1IJ49	Q571F8	Q6EQG2	Q8CS58	Q90384	Q9D1A0	Q9Y5Z9
Q21824	Q57549	Q6ER94	Q8CXF3	Q91191	Q9D975	Q9Y7F0
Q26695	Q578J2	Q6FWL5	Q8DUK3	Q92338	Q9DC60	Q9Y8D9
Q27666	Q58146	Q6G8L4	Q8DZH0	Q923X4	Q9FGS4	Q9Y9L0
Q28502	Q59081	Q6GC91	Q8E536	Q92BC5	Q9FK60	Q9Z0V5
Q2FJN4	Q59452	Q6GFZ4	Q8EPB7	Q949U7	Q9FR35	Q9Z0V6
Q2G282	Q59623	Q6GJR7	Q8F7T2	Q94BT9	Q9FWR4	Q9Z1R3
Q2QL34	Q59689	Q6HL81	Q8FQH8	Q95079	Q9H2X6	Q9Z2X8
Q2YKW2	Q5E947	Q6L140	Q8FVW4	Q95081	Q9HEY7	Q9ZKE7
Q2YVK2	Q5HF61	Q6L240	Q8GY89	Q95085	Q9HJL3	Q9ZUU2
Q39172	Q5HIR5	Q6LY19	Q8HXP8	Q95086	Q9HKX0	
Q39173	Q5HNJ2	Q6NGT4	Q8HXP9	Q95087	Q9JKY1	
Q3J6L0	Q5HRY1	Q6PBP3	Q8HXQ0	Q95088	Q9K813	
Q3KTM2	Q5JF30	Q6QPJ6	Q8HXQ1	Q95095	Q9KCJ4	
Q42564	Q5KXL9	Q6S5J6	Q8HXQ2	Q96291	Q9L3Q5	
